# The systemic immune-inflammation biomarkers in Sjögren’s disease: associations with disease activity and extraglandular manifestations in a multicentric Italian cohort

**DOI:** 10.1007/s11739-026-04394-y

**Published:** 2026-05-22

**Authors:** Stefano Stano, Venerito Vincenzo, Maria Giannotta, Fabio Cacciapaglia, Daniele Catamerò, Maria Iacovantuono, Paola Conigliaro, Benedetta Monosi, Daniele Domanico, Eduardo Urgesi, Antonio Vitale, Maria Sole Chimenti, Florenzo Iannone, Giuseppe Lopalco

**Affiliations:** 1 Rheumatology Unit - Department of Precision and Regenerative Medicine, Jonian Area (DiPReMeJ), University of Bari “Aldo Moro”, Piazza G. Cesare 11, 70124 Bari, Italy; 2 Rheumatology Unit – E.E. Regional General Hospital “F. Miulli”, Department of Medicine and Surgery, LUM “G. Degennaro”, Casamassima, Bari, Italy; 3 Rheumatology, Allergology and Clinical Immunology, Department of Systems’ Medicine, University of Rome Tor Vergata, Rome, Italy; 4 University Cardiologic Unit - Interdisciplinary Department of Medicine, Policlinico University Hospital, University of Bari “Aldo Moro”, Bari, Italy; 5 Department of Medical Sciences, Surgery and Neurosciences, Research Center of Systemic Autoinflammatory Diseases and Behçet’s Disease Clinic, University of Siena, Siena, Italy

**Keywords:** Sjögren’s disease, Extraglandular manifestations, Systemic immune-inflammation biomarkers, Disease activity

## Abstract

Sjögren's disease (SjD) is an autoimmune exocrinopathy characterized by heterogeneous extraglandular manifestations (EGM). The systemic immune-inflammation biomarkers, including the neutrophil-to-lymphocyte ratio (NLR), platelet-to-lymphocyte ratio (PLR), and systemic immune-inflammation index (SII), have been proposed as indicators of the inflammatory burden, but data in SjD remain limited. This retrospective multicenter Italian study aimed to evaluate the NLR, PLR, and SII in exocrine SjD patients and across specific EGM. Consecutive patients fulfilling the 2016 ACR/EULAR classification criteria for SjD and with available full blood count data were included. Patients were classified as having exocrine or extraglandular SjD according to the presence of specific EGM. The NLR, PLR, and SII were calculated and compared across disease phenotypes and activity levels. Among 239 SjD patients (96% female, median age 59 years), 70% presented at least one EGM. Extraglandular SjD patients had longer disease duration (*p* = 0.04) and higher frequencies of hypergammaglobulinemia (*p* = 0.05), anti-nuclear antibodies (*p* = 0.0003), anti-SSA antibodies (*p* = 0.04), and rheumatoid factor (*p* = 0.04). Overall, the median NLR, PLR, and SII did not differ between exocrine and extraglandular SjD. However, patients with arthritis showed higher NLR (*p* = 0.02), PLR (*p* < 0.05), and SII (*p* = 0.005), while patients with interstitial lung disease displayed higher SII (*p* = 0.01). Extraglandular SjD patients with moderate-to-high disease activity showed increased NLR and SII (*p* = 0.004, for both). Systemic immune-inflammation biomarkers are inexpensive, readily available and easy to calculate. The NLR, PLR, and SII are associated with specific inflammatory phenotypes and higher disease activity in Italian SjD patients. Prospective studies are warranted to confirm these preliminary findings.

**Supplementary Information:**

The online version contains supplementary material available at https://doi.org/10.1007/s11739-026-04394-y.

## Introduction

Sjögren’s disease (SjD) is a systemic autoimmune disease of unknown etiology, characterized by lymphocytic infiltration and progressive damage of the exocrine glands, chronic epithelitis and sialoadenitis [1]. The hallmark clinical manifestation of SjD is dryness, most commonly presenting as xerostomia and xerophthalmia, although any mucosal surface may be affected [2]. The extraglandular manifestations (EGM) of SjD are frequent and highly heterogeneous, with prevalence ranging from 40 to 90%, depending on definitions and cohort characteristics [3]. These EGM are commonly categorized into non-visceral manifestations, including musculoskeletal and cutaneous, and visceral manifestations, such as neurological, renal, hematological, gastrointestinal, pulmonary, and cardiovascular [4, 5].

Given this clinical heterogeneity, disease activity in SjD is commonly assessed using composite indices, including the EULAR Sjögren's Syndrome Patient-Reported Index (ESSPRI) and Disease Activity Index (ESSDAI), which capture both patient-reported symptoms and systemic disease involvement [6, 7]. However, the comprehensive evaluation of extraglandular disease remains challenging in routine clinical practice, as underscored by recent recommendations focusing on selected organ domains [8].

To date, no single specific biomarker of SjD has been validated for disease activity assessment in clinical practice [9, 10]. Conventional inflammatory markers, such as complement fractions, β2-microglobulin, C-reactive protein (CRP), and erythrocyte sedimentation rate (ESR) levels, show limited sensitivity and specificity in reflecting disease activity [11]. In this context, systemic immune-inflammation biomarkers, such as the neutrophil-to-lymphocyte ratio (NLR), platelet-to-lymphocyte ratio (PLR), and systemic immune-inflammation index (SII), emerged as potential indicators of the inflammatory burden [12–14], easily derived from routine full blood count (FBC) parameters [15]. These indices have been reported to be increased in SjD patients compared with healthy controls and to correlate with disease activity and circulating pro-inflammatory cytokines, such as interleukin (IL)-6, IL-7 and TNF-α [16, 17]. However, data on the expression of systemic immune-inflammation biomarkers across different SjD clinical subsets remain limited.

This study aimed to assess the systemic immune-inflammation biomarkers in a large Italian cohort of SjD patients, comparing individuals with isolated exocrine involvement and those presenting specific extraglandular manifestations. A comparison of these biomarkers in SjD patients with high-moderate versus low disease activity as defined with ESSDAI, and across different ESSPRI levels, was also performed.

## Methods

### Study population and design

This multicenter retrospective observational study included consecutive patients who were treated from January 2023 to January 2025 at three referral SjD Units in Italy (Bari, Rome, Siena). Eligible patients fulfilled the 2016 American College of Rheumatology/European Alliance of Associations for Rheumatology (ACR/EULAR) classification criteria for SjD, and had a complete FBC assessment performed within 1 month from the scheduled visit and assessed at the time of the clinical evaluation, allowing calculation of the systemic immune-inflammatory biomarkers [18]. The diagnosis of other concomitant autoimmune diseases as rheumatoid arthritis (RA), psoriatic arthritis (PsA), or any connective tissue disease (CTD), meeting the specific classification criteria, was considered an exclusion criterion.

Patients were categorized according to their clinical phenotype: 1) “exocrine SjD” was defined by the presence of sicca symptoms and/or exocrine gland swelling in the absence of any systemic involvement; 2) “extraglandular SjD” was defined by the presence of at least one EGM demonstrated at the time of evaluation or documented in the clinical history, including lymphoma, interstitial lung disease (ILD), renal or neurological involvement, arthritis, arthralgia, cryoglobulinemia, or cutaneous manifestations.

Data were retrospectively collected from medical records using a shared study protocol and standardized case reports forms, and subsequently pooled in a centralized database. Comparisons were performed between patients with exocrine and extraglandular SjD. The study was conducted in accordance with the Declaration of Helsinki and was approved by the local ethics Committees of the participating centers, with Bari Policlinico Hospital acting as the coordinating center (protocol n° 5277). Written informed consent, including authorization for the processing of personal data, was obtained from all participants. The study was reported in accordance with the Strengthening the Reporting of Observational Studies in Epidemiology (STROBE) guidelines [19].

### Clinical manifestations and disease activity assessment

Clinical manifestations and organ involvement attributable to SjD were assessed using validated disease-specific composite indices. Patient-reported symptoms were evaluated using ESSPRI, which captures pain, fatigue, and dryness. Systemic disease activity was assessed using ESSDAI, encompassing multiple organ-specific domains [20]. Objective assessment of glandular involvement included evaluation of ocular dryness by the Schirmer test. Minor salivary gland biopsy was performed in seronegative patients to confirm the diagnosis and, when clinically indicated, according to standard clinical practice [21]. The presence of ILD was suspected in the presence of typical symptoms, auscultation findings, or abnormal pulmonary function tests (PFTs), including forced vital capacity (FVC) and diffusing lung capacity for carbon monoxide (DLCO). High-resolution computed tomography (HRCT) of the lungs was performed to confirm the diagnosis of ILD and, when clinically indicated, the case was evaluated by a multidisciplinary team including rheumatologists, pneumologists, and radiologists. The presence of comorbidities, including hypertension, osteoporosis, fibromyalgia, diabetes, and mood disorders, was recorded based on medical history and available clinical documentation.

### Laboratory assessments and treatments

The laboratory data, collected during the clinical assessment, included the FBC, serum protein electrophoresis, cryoglobulins, β2-microglobulin, ESR, CRP, serum immunoglobulins (Ig) A, G, and M, lactate dehydrogenase (LDH), and complement fractions C3 and C4. Metabolic parameters, including total cholesterol, high-density lipoprotein (HDL), low-density lipoprotein (LDL), triglycerides, and fasting morning blood glucose levels, were also collected as part of the routine clinical evaluation. The autoantibody assessment included the anti-nuclear antibodies (ANA), anti-Ro/SSA, anti-La/SSB, and rheumatoid factor (RF). Reported treatments included corticosteroids (CS), conventional synthetic (cs) disease-modifying antirheumatic drugs (DMARDs), biologic (b) DMARDs, and symptomatic therapies, including pilocarpine, myorelaxants, and antidepressants. Systemic immune-inflammatory biomarkers (NLR, PLR and SII) were calculated using FBC-derived values available at the time of clinical evaluation and compared between patients with exocrine SjD and those with EGM.

## Objectives

Primary aim:

- To compare the systemic immune-inflammation biomarkers NLR, PLR, and SII between patients with exocrine SjD and those with extraglandular involvement.

Secondary aims:

- To evaluate the systemic immune-inflammation biomarkers across specific EGM of SjD.
- To explore the relationship between the systemic immune-inflammation biomarkers and SjD disease activity, as assessed by ESSDAI and ESSPRI.

## Statistical analysis

Continuous variables were reported as mean and standard deviation (SD), median and interquartile range (IQR), or number and percentage, as appropriate. Categorical variables were expressed as absolute numbers and percentages. The D’Agostino-Pearson test was employed to assess the distribution of data. Comparisons between groups were performed using the paired t-test or the Mann–Whitney *U* test for continuous variables, while the Chi-square test or Fischer exact test were used for categorical variables. A two-tailed *p*-value < 0.05 was considered statistically significant. Missing data were handled using a complete-case approach without imputation and clearly reported in tables. All statistical analyses were performed using GraphPad Prism (v.10) software.

## Results

### Clinical and demographic characteristics

A total of 239 SjD patients (96% female, median (IQR) age 59 (51–69) years) were included in the analysis; among them 168 (70%) presented at least one EGM (Table 1).

**Table 1:** Demographics and clinical characteristics of patients with exocrine vs extraglandular Sjögren’s disease

Characteristics	SjD patients (*n.* 239)	Exocrine SjD (*n.* 71)	Extraglandular SjD (*n.* 168)	*p*-value	Missing data
*Clinical characteristics*					
Female, n. (%)	229 (95.8)	67 (94.4)	162 (96.7)	0.49	0
Age (years), median (IQR)	59 (51–69)	60 (51–69)	59 (52–69)	0.76	1
Disease duration (months), median (IQR)	120 (72–192)	96 (59–168)	132 (80–197)	**0.04**	22
Osteoporosis, n. (%)	79 (33)	22 (31)	57 (33.9)	0.66	0
T2DM, n. (%)	13 (5.6)	2 (2.9)	11 (6.7)	0.35	7
Fibromyalgia, n. (%)	71 (29.7)	15 (21.1)	56 (33.3)	0.06	0
MADD, n. (%)	22 (9.2)	10 (14.1)	12 (7.1)	0.09	0
Dryness, n. (%)	220 (92)	67 (94.4)	153 (91.1)	0.45	0
Exocrine glands swelling, n. (%)	28 (11.7)	4 (5.6)	24 (14.3)	0.08	0

*IQR* interquartile range, *MADD* mixed anxiety–depressive disorder, *n* number, *T2DM* type 2 diabetes mellitus. Bold value indicates a statistically significant difference (p < 0.05).

The median (IQR) disease duration was 10 (6–16) years, and this was significantly longer in the extraglandular group than in those with exocrine disease (*p* = 0.04), while age and sex distributions were similar between groups. Fibromyalgia tended to be more frequent in extraglandular SjD patients, although the difference did not reach statistical significance (*p* = 0.06). Conversely, mood disorders were more commonly reported in patients with exocrine SjD, again without a significant difference between groups (*p* = 0.09). Other comorbidities were similarly distributed between the two groups. In the entire cohort, dryness was the most frequently reported symptom (92%), while exocrine glands swelling was observed in 12% of cases and showed a trend toward higher prevalence in the extraglandular SjD group (*p* = 0.08). Among EGM, arthralgias were the most common (46%), followed by cutaneous involvement (18%), arthritis (12%), peripheral neuropathy (12%), cryoglobulinemia (10%), ILD (10%), renal involvement (4%), lymphoma (3%), and central nervous system (CNS) involvement (2%), as shown in Supplementary Fig. 1.

### Laboratory features according to disease phenotype

Extraglandular SjD patients showed a trend toward higher inflammatory markers, with more frequent elevations of ESR (*p* = 0.10) and CRP (*p* = 0.09), and significantly higher platelet counts compared with exocrine SjD patients (*p* = 0.02). Total leukocyte, neutrophil, and lymphocyte counts did not differ between groups, and overall FBC parameters were within the normal range, as described in Table 2.

**Table 2:** Laboratory findings in Sjögren’s disease patients with exocrine or extraglandular manifestations

Laboratory parameters	SjD patients (*n.* 239)	Exocrine SjD (*n.* 71)	Extraglandular SjD (*n.* 168)	*p*-value	Missing data
ESR increase (> 20 mg/L), n. (%)	76 (31.8)	17 (23.9)	59 (35.1)	0.10	0
CRP increase (> 5 mg/L), n. (%)	31 (13)	5 (7)	26 (15.5)	0.09	0
Leukocytes (N*10^9^/L), median (IQR)	5.36 (4.39–6.38)	5.30 (4.36–6.03)	5.50 (4.41–6.65)	0.16	0
Neutrophils (N*10^9^/L), median (IQR)	2.83 (2.32–3.86)	2.83 (2.40–3.52)	2.85 (2.29–3.95)	0.83	0
Lymphocytes (N*10^9^/L), median (IQR)	1.70 (1.31–2.19)	1.72 (1.39–2.10)	1.70 (1.30–2.25)	0.69	0
Platelets (N*10^9^/L), median (IQR)	231 (195–275)	223 (187–253)	238 (198–292)	**0.02**	0
NLR, median (IQR)	1.70 (1.27–2.43)	1.63 (1.31–2.23)	1.75 (1.25–2.56)	0.83	0
PLR, median (IQR)	140 (102–177)	133 (100–167)	142 (103–183)	0.19	0
SII (N*10^9^/L), median (IQR)	396 (283–586)	369 (272–519)	417 (291–592)	0.18	0
Hypocomplementemia, n. (%)	68 (28.4)	17 (23.9)	51 (30.4)	0.31	0
C3 level (mg/dL), median (IQR)	109 (95–127)	111 (96–128)	109 (94–127)	0.71	39
C4 level (mg/dL), median (IQR)	24 (18–30)	24 (18–31)	24 (17–30)	0.81	40
LDH increase (> 300 U/L), n. (%)	53 (22.4)	11 (15.5)	42 (25.3)	0.09	3
Hypergammaglobulinemia, n. (%)	89 (37.4)	20 (28.2)	69 (41.3)	**<0.05**	1
Hypogammaglobulinemia, n. (%)	18 (7.6)	4 (5.6)	14 (8.4)	0.60	1
β2-microglobulin (mg/dL), median (IQR)	2.3 (1.7–2.8)	2.1 (1.8–2.5)	2.3 (1.7–2.9)	0.39	69
IgA level (mg/dL), median (IQR)	226 (155–299)	232 (169–303)	226 (155–296)	0.77	37
IgG level (mg/dL), median (IQR)	1298 (1005–1738)	1200 (873–1610)	1300 (1008–1741)	0.67	36
IgM level (mg/dL), median (IQR)	103 (61–165)	88 (51–144)	106 (66–170)	0.38	38
Total cholesterol (mg/dL), median (IQR)	191 (166–219)	207 (170–222)	183 (165–214)	0.14	54
HDL cholesterol (mg/dL), median (IQR)	62 (51–78)	65 (52–85)	61 (50–76)	0.18	59
LDL cholesterol (mg/dL), median (IQR)	105 (87–126)	108 (83–135)	105 (88–126)	0.76	54
Triglycerides (mg/dL), median (IQR)	84 (60–111)	73 (56–113)	87 (65–111)	0.26	54
Fasting glycemia (mg/dL), median (IQR)	86 (79–95)	89 (82–93)	85 (78–96)	0.70	46
ANA positive, n. (%)	187 (79.9)	45 (65.2)	142 (86.1)	**<0.01**	5
Anti-SSA positive n. (%)	165 (70.5)	42 (60.9)	123 (74.5)	**0.04**	5
Anti-SSB positive, n. (%)	84 (35.9)	21 (30.4)	63 (38.2)	0.26	5
RF positive, n. (%)	47 (20.1)	8 (11.6)	39 (23.6)	**0.04**	5

*ANA* anti-nuclear antibodies, *CRP* C-reactive protein, *C3* complement component 3, *C4* complement component 4, *ESR* erythrocyte sedimentation rate, *FM* fibromyalgia, *HDL* high-density lipoprotein, *Ig* immunoglobulins, *IQR* interquartile range, *LDH* lactate dehydrogenase, *LDL* low-density lipoprotein, *n* number, *NLR* neutrophil-to-lymphocyte ratio, *PLR* platelet-to-lymphocyte ratio, *RF* rheumatoid factor, *SII* systemic immune-inflammation index, *SjD* Sjögren’s disease, *SSA* Sjögren’s syndrome-related antigen A, *SSB* Sjögren's syndrome-related antigen B. Bold values indicate statistically significant differences (p-value < 0.05).

The median (IQR) NLR was 1.70 (1.27–2.43) and did not differ between exocrine and extraglandular SjD. Similarly, the median (IQR) PLR was 140 (102–177), and the median (IQR) SII was 396 (283–586), with no significant differences between groups. Hypocomplementemia was observed in 28% of patients, with comparable C3 and C4 levels; LDH was increased in 22% of patients and showed a non-significant trend toward higher levels in those with EGM (*p* = 0.09). Hypergammaglobulinemia was frequently detected (37%) and occurred significantly more often in extraglandular SjD patients (*p* = 0.05), while hypogammaglobulinemia was uncommon (< 8%) and similarly distributed between groups. Serum β2-microglobulin, IgA, IgG and IgM levels were within the normal range and did not differ according to disease phenotype. Lipid profile parameters were comparable between groups, with mildly increased LDL cholesterol, normal total cholesterol and fasting glucose, and preserved HDL cholesterol and triglyceride levels overall.

Regarding the autoimmune profile, ANA were detected in 80% of patients, anti-SSA in 70%, anti-SSB in 36%, and RF in 20%. Extraglandular SjD patients more frequently displayed ANA positivity (*p* = 0.0003), anti-SSA antibodies (*p* = 0.04) and RF (*p* = 0.04).

### Disease activity, patient-reported outcomes, and treatments

Pulmonary function test showed overall preserved respiratory function, as the median predicted FVC and DLCO were within the normal range and comparable between exocrine and extraglandular SjD patients. The Schirmer test was positive in 72% of the cases, while a minor salivary gland biopsy focus score ≥ 1 was observed in 51% of patients (Table 3).

**Table 3:** Disease activity assessment and treatments in exocrine and extraglandular Sjögren’s disease patients

Disease assessment	SjD patients (*n.* 239)	Exocrine SjD (*n.* 71)	Extraglandular SjD (*n.* 168)	*p*-value	Missing data
FVC (%) last, median (IQR)	107 (96–118)	102 (89–118)	110 (98–118)	0.40	37
DLCO (%) last, median (IQR)	75 (63–93)	77 (60–103)	74 (63–91)	0.58	34
Schirmer test positive, n. (%)	127 (72.2)	40 (80)	87 (69)	0.14	33
Focus score positive, n. (%)	55 (50.9)	21 (56.8)	34 (47.9)	0.38	131
ESSDAI, median (IQR)	1 (0–2)	0 (0–2)	1 (0–3)	0.17	2
ESSPRI, median (IQR)	6.3 (4–7.7)	5.8 (3.7–7.2)	6.7 (4.8–8)	0.07	23
Fatigue VAS (0–10), median (IQR)	7 (5–8)	6 (2.2–8)	7 (5–8)	**0.02**	23
Dryness VAS (0–10), median (IQR)	7 (5–8)	7 (5–8)	6 (5–8)	0.52	23
Pain VAS (0–10), median (IQR)	6 (2–8)	4.5 (0–7.7)	7 (5–8)	**<0.01**	23
csDMARD, n. (%)	206 (86.2)	62 (87.3)	144 (85.7)	0.74	0
bDMARD, n. (%)	19 (8)	2 (2.8)	17 (10.2)	0.07	0
CS, n. (%)	95 (39.7)	22 (31)	73 (43.4)	0.07	0
CS PDNeq (mg/day), median (IQR)	5 (2.5–5)	2.9 (2.5–5)	5 (2.5–5)	0.06	0
Pilocarpine, n. (%)	98 (41.2)	37 (52.1)	61 (36.5)	**0.02**	1
Antidepressants, n. (%)	45 (18.8)	15 (21.1)	30 (17.9)	0.55	0
Myorelaxants, n. (%)	80 (33.5)	16 (22.5)	64 (38.1)	**0.02**	0

*bDMARDs* biologic disease-modifying antirheumatic drugs, *CS* corticosteroids, *csDMARD* conventional synthetic disease-modifying antirheumatic drugs, *DLCO* diffusing capacity of carbon monoxide, *ESSDAI* EULAR Sjögren syndrome disease activity index, *ESSPRI* EULAR Sjögren Syndrome Patient-Reported Index, *FM* fibromyalgia, *FVC* forced vital capacity, *HRCT* high-resolution computed tomography, *IQR* interquartile range, *n* number, *PDNeq* prednisone equivalent, *SjD* Sjögren’s disease, *VAS* visual analog scale. Bold values indicate statistically significant differences (p < 0.05).

Concerning treatments, csDMARDs were prescribed in 86% of patients, while bDMARDs were used in 8% of the cases. Ongoing CS therapy was reported in 40% of patients, at a median (IQR) daily dose of 5 (2.5–5) mg. Pilocarpine and myorelaxants were administered in 41% and 19% of patients, respectively, and were significantly more common in the exocrine SjD group (*p* = 0.02, for both). Conversely, CS and bDMARDs tended to be more frequently prescribed in patients with extraglandular involvement, although these differences did not reach statistical significance (*p* = 0.07, for both).

**Fig. 1:**
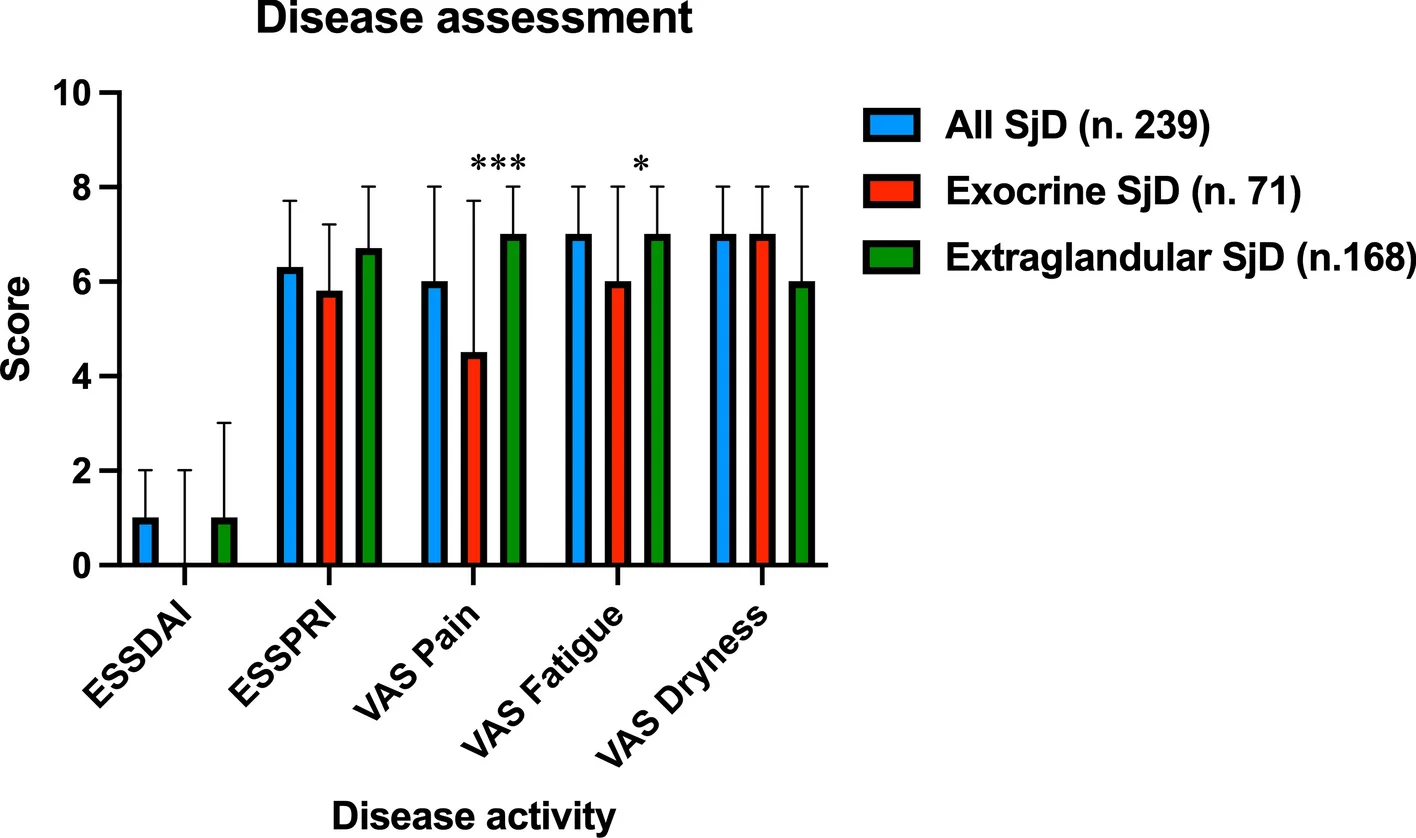
Disease activity assessments in exocrine and extraglandular Sjögren disease patients. *ESSDAI* EULAR Sjögren syndrome disease activity index, *ESSPRI* EULAR Sjögren syndrome patient-reported index, *SjD* Sjögren’s disease, *VAS* visual analog scale, *p-value < 0.05 vs exocrine SjD, **p-value < 0.01 vs exocrine SjD, ***p-value < 0.001 vs exocrine SjD

Regarding disease assessment, the median (IQR) ESSDAI was 1 (0–2) and did not differ between groups. In contrast, the median (IQR) ESSPRI was 6 (4–8), and tended to be higher in patients with EGM (*p* = 0.07). Specifically, fatigue (*p* = 0.02) and pain (*p* = 0.0006) scores were significantly higher in the extraglandular SjD group (Fig. 1).

### Systemic immune-inflammation biomarkers

Systemic immune-inflammation biomarkers did not differ between exocrine and extraglandular SjD patients when analyzed overall. However, when stratifying extraglandular SjD according to specific EGM, relevant differences emerged compared with the exocrine phenotype. Patients with ILD showed a trend toward higher NLR (*p* = 0.08) and significantly higher SII (*p* = 0.01). Similarly, the presence of arthritis was associated with significantly higher NLR (*p* = 0.02), PLR (*p* < 0.05) and SII (*p* = 0.005). A detailed description of systemic immune-inflammation biomarkers according to each EGM is provided in Supplementary Table 1.

**Fig. 2:**
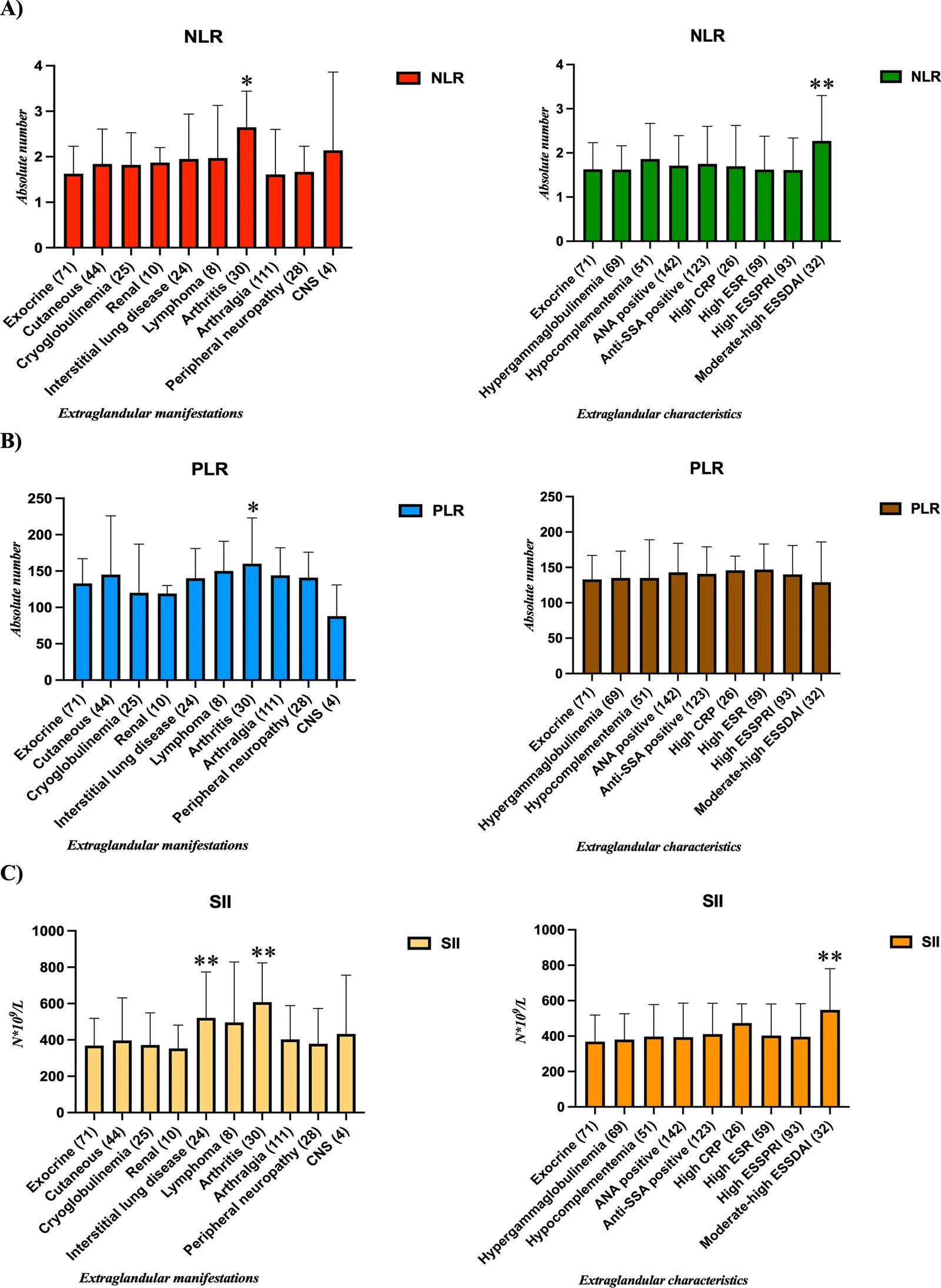
The systemic immune-inflammation biomarkers, including NLR (**A**), PLR (**B**), and SII (**C**) in exocrine Sjögren disease patients and in different extraglandular manifestations and characteristics. *ANA* anti-nuclear antibodies, *CNS* central nervous system, *CRP* C-reactive protein, *EGM* extraglandular manifestations, *ESR* erythrocyte sedimentation rate, *ESSDAI* EULAR Sjögren syndrome disease activity index, *ESSPRI* EULAR Sjögren syndrome patient-reported index, *NLR* neutrophil-to-lymphocyte ratio, *PLR* platelet-to-lymphocyte ratio, *SII* systemic immune-inflammation index, *SjD* Sjögren’s disease, *SSA* Sjögren’s syndrome-related antigen A, *p-value < 0.05 vs exocrine SjD; **p-value < 0.01 vs exocrine SjD; ***p-value < 0.001 vs exocrine SjD

No significant differences in NLR, PLR, or SII were observed among extraglandular SjD patients stratified by hypogammaglobulinemia, hypocomplementemia, ANA or anti-SSA positivity, elevated CRP or ESR, or high ESSPRI scores, when compared with exocrine SjD patients. In contrast, extraglandular SjD patients with moderate-to-high systemic disease activity, defined by higher ESSDAI scores, displayed significantly increased NLR and SII (*p* = 0.004, for both), as shown in Fig. 2.

## Discussion

In this Italian multicentric real-life cohort, we investigated the systemic immune-inflammation biomarkers NLR, PLR, and SII across different clinical phenotypes of SjD, confirming a heterogenous expression of these indices in relation to specific EGM and disease activity. To the best of our knowledge, this represents the largest study evaluating these inflammatory biomarkers across a broad spectrum of SjD systemic manifestations.

The presence of EGM was observed in approximately 70% of patients, with comparable sex and age distribution, but longer disease duration in extraglandular SjD. This was coherent with data from large international cohorts, despite significant variability observed in smaller studies, also explained by differences in patient selection, definitions of systemic involvement, and geo-epidemiological factors [22, 23].

From a laboratory perspective, exocrine and extraglandular SjD patients showed comparable inflammatory and metabolic profiles. However, extraglandular SjD patients had a significantly higher prevalence of hypergammaglobulinemia and autoantibodies including ANA, anti-SSA, and RF. This finding is consistent with previous studies linking EGM to heightened B-cell activation, with anti-SSA antibodies emerging as one of the strongest predictors of systemic involvement [24–26].

Overall, the NLR, PLR, and SII did not differ between exocrine and extraglandular SjD. However, relevant differences emerged across specific systemic manifestations and disease activity: higher NLR, PLR, and SII were observed in patients with inflammatory arthritis, higher NLR in those with ILD, and increased PLR and SII in patients with moderate-to-high ESSDAI. The associations between systemic immune-inflammation biomarkers and SjD disease activity was also reported by Kilic et al., showing higher NLR, PLR, and SII levels in SjD patients compared with healthy controls, with SII increasing in moderate-to-severe disease activity, in line with our findings [27]. Other studies confirmed significant correlations between PLR, SII, NLR, and salivary gland pathology [28, 29]. While direct evidence regarding these biomarkers in SjD-related arthritis is absent, data from RA and PsA cohorts consistently showed elevated SII values associated with disease activity, joint inflammation, and adverse outcomes, including mortality, suggesting potential prognostic relevance [30–35]. In our study, patients with other concomitant autoimmune diseases including RA, PsA, and CTD were excluded as described in the methods section, further supporting the existence of a direct link between SjD-related arthritis and increased NLR, PLR, and SII. The systemic immune-inflammation biomarkers have also been explored in CTD-associated ILD. Previous studies reported higher NLR, PLR and SII in SjD-ILD and negative correlations with pulmonary function parameters, including DLCO [36–38].

The highest NLR values were observed in patients with lymphoma or central nervous system involvement; however, the low prevalence of these manifestations limited the strength of the statistical analysis. Interestingly, an elevated NLR has been recently associated with poor prognosis in malignancies and immune-mediated neurological disorders, and emerging data suggest a potential role in predicting neurological involvement in SjD [39–41]. Conversely, despite previous reports linking increased NLR and PLR to SjD-related cutaneous vasculitis, we did not observe significant differences in patients with cutaneous involvement [42].

## Limitations and future perspectives

The limitations of this study include the retrospective design, lacking a longitudinal validation of data. The real-life setting may have resulted in missing data and selection bias. Data regarding the salivary glands’ ultrasonography, histology and the salivary flow assessment presented a significant number of missing variables and were not included in this analysis. The effects of concomitant treatments and comorbidities may have affected the levels of the systemic immune-inflammation biomarkers, but these were not considered in the statistical analysis of data. Finally, some EGM of SjD were rare, limiting the ability to detect significant differences compared with the exocrine SjD group.

Our findings support a differential expression of systemic immune-inflammation biomarkers across specific SjD EGM and higher ESSDAI. Prospective studies and randomized clinical trials should evaluate the potential of these biomarkers to predict the development of EGM or disease progression in SjD patients.

## Conclusions

Systemic immune-inflammation biomarkers are inexpensive, readily available, and easy to calculate. 

The presence of arthritis was associated with increased PLR, NLR, and SII; ILD was linked with higher SII; finally, a moderate–high ESSDAI was associated with higher NLR and SII.

These preliminary findings suggest that NLR, PLR, and SII may identify SjD patients with higher inflammatory burden and specific extraglandular manifestations.

## Supplementary Information

Below is the link to the electronic supplementary material.

Supplementary file1 Frequency histogram of extraglandular manifestations (EGM) observed in Sjögren’s disease patients. Abbreviations: CNS, central nervous system; ILD, interstitial lung disease; n, number (TIFF 135945 KB)

Supplementary file2 (DOCX 26 KB)

## Data Availability

The data that support the findings of this study are available from the corresponding author upon reasonable request.
