# Supplementary material for: The systemic immune-inflammation biomarkers in Sjögren’s disease: associations with disease activity and extraglandular manifestations in a multicentric Italian cohort
Source: Intern Emerg Med. 2026 May 22;21(6):2109–19. doi: 10.1007/s11739-026-04394-y (PMC13582351; doi:10.1007/s11739-026-04394-y)
Supplement: Supplementary file 1 [file 11739_2026_4394_MOESM2_ESM.docx]

**Supplementary Table 1.** Systemic immune-inflammation biomarkers in Sjögren’s disease patients. The NLR, PLR and SII of exocrine SjD patients are compared to those of patients with different extraglandular manifestations (EGM) and clinical characteristics. The p-value for each comparison is reported.

| **SjD manifestations (n)** | **NLR** | **p-value** | **PLR** | **p-value** | **SII (N*10^9^/L)** | **p-value** |
| --- | --- | --- | --- | --- | --- | --- |
| **Exocrine (71)** | 1.63 (1.31-2.23) | NA | 133 (100-167) | NA | 369 (272-519) | NA |
| ***Extraglandular manifestations*** | | | | | | |
| **Cutaneous (44)** | 1.84 (1.32-2.61) | 0.49 | 145 (99-226) | 0.10 | 397 (269-631) | 0.22 |
| **Cryoglobulinemia (25)** | 1.82 (1.22-2.53) | 0.90 | 120 (89-187) | 0.85 | 372 (288-549) | 0.99 |
| **Renal (10)** | 1.87 (1.46-2.20) | 0.74 | 119 (104-130) | 0.39 | 353 (278-481) | 0.73 |
| **Interstitial lung disease (24)** | 1.95 (1.67-2.94) | 0.08 | 140 (108-181) | 0.42 | 521 (339-774) | **0.01** |
| **Lymphoma (8)** | 1.97 (1.41-3.13) | 0.45 | 150 (116-191) | 0.25 | 496 (366-829) | 0.15 |
| **Arthritis (30)** | 2.65 (1.44-3.44) | **0.02** | 160 (112-223) | **<0.05** | 608 (338-824) | **0.005** |
| **Arthralgia (111)** | 1.61 (1.21-2.60) | 0.65 | 144 (101-182) | 0.22 | 403 (282-589) | 0.40 |
| **Peripheral neuropathy (28)** | 1.67 (1.34-2.23) | 0.91 | 141 (120-176) | 0.37 | 379 (297-573) | 0.50 |
| **CNS (4)** | 2.14 (0.99-3.86) | 0.99 | 88 (66-131) | 0.10 | 433 (244-756) | 0.71 |
| ***Clinical characteristics*** | | | | | | |
| **Hypergammaglobulinemia (69)** | 1.62 (1.20-2.16) | 0.43 | 135 (101-173) | 0.51 | 380 (250-526) | 0.98 |
| **Hypocomplementemia (51)** | 1.86 (1.12-2.67) | 0.96 | 135 (102-189) | 0.31 | 397 (265-578) | 0.62 |
| **ANA positive (142)** | 1.71 (1.22-2.39) | 0.79 | 143 (106-184) | 0.16 | 394 (283-586) | 0.37 |
| **Anti-SSA positive (123)** | 1.75 (1.27-2.60) | 0.72 | 141 (104-179) | 0.20 | 411 (290-585) | 0.21 |
| **High CRP (26)** | 1.70 (1.24-2.62) | 0.98 | 146 (100-166) | 0.64 | 474 (254-582) | 0.37 |
| **High ESR (59)** | 1.62 (1.27-2.38) | 0.76 | 147 (98-183) | 0.28 | 403 (257-581) | 0.46 |
| **High ESSPRI (93)** | 1.61 (1.22-2.34) | 0.66 | 140 (101-181) | 0.36 | 396 (299-583) | 0.42 |
| **Moderate-high ESSDAI (32)** | 2.27 (1.70-3.30) | **0.004** | 129 (99-186) | 0.81 | 548 (350-780) | **0.004** |

Abbreviations: ANA, anti-nuclear antibodies; CNS, central nervous system; CRP, C-Reactive protein; ESR, erythrocyte sedimentation rate; ESSDAI, EULAR Sjögren Syndrome disease activity index; ESSPRI, EULAR Sjögren Syndrome patient reported index; n. number; NA, not assessed; NLR, neutrophil-to-lymphocyte ratio; PLR, platelet-to-lymphocyte ratio; SII, systemic immune-inflammation index; SjD, Sjögren’s disease; SSA, Sjögren’s-syndrome-related antigen A;
